# Ligand-induced Epitope Masking: DISSOCIATION OF INTEGRIN α5β1-FIBRONECTIN COMPLEXES ONLY BY MONOCLONAL ANTIBODIES WITH AN ALLOSTERIC MODE OF ACTION*[Fn FN2]

**DOI:** 10.1074/jbc.M116.736942

**Published:** 2016-08-02

**Authors:** A. Paul Mould, Janet A. Askari, Adam Byron, Yoshikazu Takada, Thomas A. Jowitt, Martin J. Humphries

**Affiliations:** From the ‡ Biomolecular Analysis Core Facility and; § Wellcome Trust Centre for Cell-Matrix Research, Faculty of Life Sciences, University of Manchester, Manchester M13 9PT, United Kingdom,; the ¶ Edinburgh Cancer Research UK Centre, Institute of Genetics and Molecular Medicine, University of Edinburgh, Edinburgh EH4 2XR, Scotland, United Kingdom, and; the ‖ Department of Vascular Biology, VB-1, The Scripps Research Institute, La Jolla, California 92037

**Keywords:** allosteric regulation, antibody, cell adhesion, fibronectin, integrin, epitope masking, therapeutics

## Abstract

We previously demonstrated that Arg-Gly-Asp (RGD)-containing ligand-mimetic inhibitors of integrins are unable to dissociate pre-formed integrin-fibronectin complexes (IFCs). These observations suggested that amino acid residues involved in integrin-fibronectin binding become obscured in the ligand-occupied state. Because the epitopes of some function-blocking anti-integrin monoclonal antibodies (mAbs) lie near the ligand-binding pocket, it follows that the epitopes of these mAbs may become shielded in the ligand-occupied state. Here, we tested whether function-blocking mAbs directed against α5β1 can interact with the integrin after it forms a complex with an RGD-containing fragment of fibronectin. We showed that the anti-α5 subunit mAbs JBS5, SNAKA52, 16, and P1D6 failed to disrupt IFCs and hence appeared unable to bind to the ligand-occupied state. In contrast, the allosteric anti-β1 subunit mAbs 13, 4B4, and AIIB2 could dissociate IFCs and therefore were able to interact with the ligand-bound state. However, another class of function-blocking anti-β1 mAbs, exemplified by Lia1/2, could not disrupt IFCs. This second class of mAbs was also distinguished from 13, 4B4, and AIIB2 by their ability to induce homotypic cell aggregation. Although the epitope of Lia1/2 was closely overlapping with those of 13, 4B4, and AIIB2, it appeared to lie closer to the ligand-binding pocket. A new model of the α5β1-fibronectin complex supports our hypothesis that the epitopes of mAbs that fail to bind to the ligand-occupied state lie within, or very close to, the integrin-fibronectin interface. Importantly, our findings imply that the efficacy of some therapeutic anti-integrin mAbs could be limited by epitope masking.

## Introduction

Integrins are a large family of α- and β-heterodimeric transmembrane receptors that mediate many cell-cell and cell-matrix interactions (1). These interactions are indispensable for normal development, cell survival, and organ and immune system function. In many disorders, aberrant integrin function plays a role in initiating, maintaining, or exacerbating the disease process. Several integrin family members are important targets for therapy in autoimmunity, thrombosis, fibrosis, and cancer, including α4β1, α4β7, αIIbβ3, α5β1, αVβ3, αVβ6, and αVβ8 (2, 3). Currently, most efficacious integrin-based therapies involve the use of humanized mAbs that block the ligand-binding site of the integrin (4–6), and these drugs have combined annual sales of over $2 billion (2). In contrast, some mAb-based therapies have failed in clinical trials (7–9).

Ligand binding to integrins takes place in the so-called “headpiece” domain (10, 11). This region includes the seven-bladed β-propeller domain of the α subunit and the βI and hybrid domains of the β subunit. Loops on the upper surface of the β-propeller and the top face of the βI domain form the ligand-binding pocket (12–17). The metal ion-dependent adhesion site (MIDAS)^3^ on the top of the βI domain is an essential site for binding to ligands that contain a carboxyl group (such as the RGD peptide sequence) (11, 12, 17, 18). Some integrin ligands contain a second site for integrin binding, such as the Pro-His-Ser-Arg-Asn (PHSRN) synergy sequence in fibronectin (19).

Integrins can exist in different conformational states, and high affinity ligand binding requires an opening of the headpiece (20–22). Headpiece opening involves shifts of the α1 and α7 helices in the βI domain and an outward swing of the hybrid domain away from the β-propeller (11, 23–26). The epitopes of function-blocking anti-integrin α subunit antibodies lie in loops in blades 1–3 of the propeller, overlapping with or close to the loops involved in ligand recognition (27–31). The epitopes of nearly all inhibitory anti-β subunit mAbs lie within the βI domain (11, 21, 32–34). In the integrin β1 subunit, these epitopes involve residues in the α2 helix of βI (17, 18, 30), and most of these mAbs appear to block integrin function by preventing an inward movement of the adjacent α1 helix (17, 23). Hence, many of these anti-β1 mAbs have an allosteric mode of action.

Recently, we showed that RGD-containing ligand-mimetic inhibitors of integrins are unable to dissociate pre-formed IFCs (35), a feature that could have contributed to the failure of compounds such as cilengitide in cancer treatment (36, 37). Our observations provided evidence that the RGD-binding pocket is obscured in the macromolecular ligand-occupied state, *i.e.* integrin residues involved in ligand recognition become buried in the integrin-fibronectin interface. Because the residues that form the epitopes of some function-blocking mAbs lie very close to the ligand-binding pocket, it follows that the epitopes of these mAbs may become obscured in the ligand-occupied state. Hence, these mAbs could fail to bind to, or cause disruption of, IFCs.

Here, we have tested the ability of many different mAbs directed against the α and β subunits of the fibronectin receptor α5β1 to bind to and disrupt IFCs. We show that function-blocking antibodies directed against the α5 subunit fail to dissociate these complexes, suggesting that the epitopes of these mAbs are masked. In contrast, most function-blocking antibodies directed against the β1 subunit can disrupt IFCs, demonstrating that the epitopes of these antibodies are still accessible in the ligand-bound state. Additionally, we map the epitope of the unusual anti-β1 mAb Lia1/2, which, like the anti-α5 subunit mAbs, fails to dissociate integrin-ligand complexes, and we provide evidence that its epitope partly overlaps with the ligand-binding pocket. Our results suggest that epitopes that are spatially close to residues involved in ligand recognition become obscured in the IFC. An important corollary of these data is that the effectiveness of some therapeutic mAbs could be limited by their epitopes becoming masked in ligand-occupied integrins.

## Results

### 

#### 

##### Function-blocking Anti-α5 mAbs Cannot Disrupt Pre-formed α5β1-Fibronectin Complexes

**FIGURE 1.**
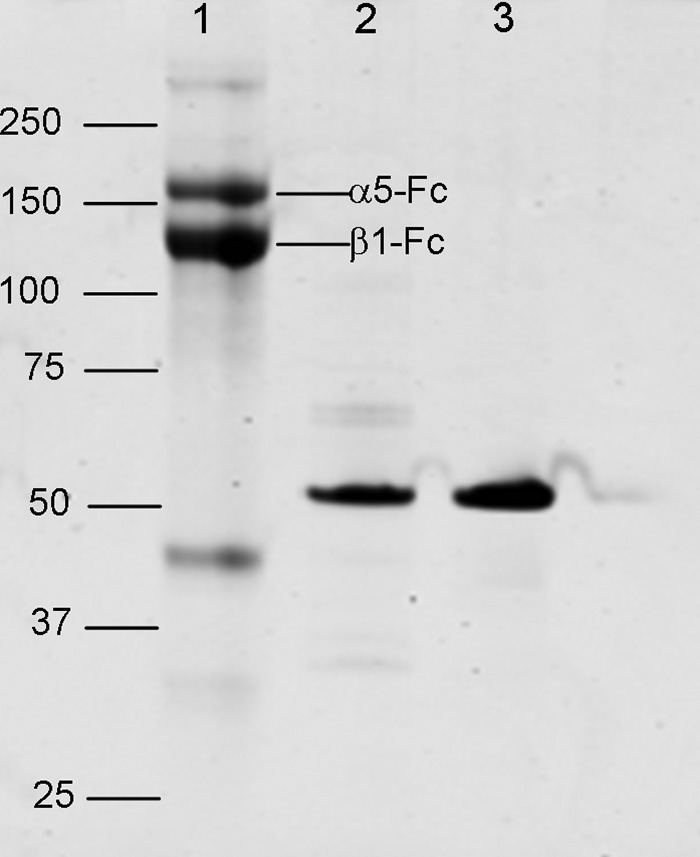
**SDS-PAGE of the recombinant integrin and fibronectin fragments used in these experiments.** Samples were run on a 4–12% gel under reducing conditions. *Lane 1,* α5β1-Fc; *lane 2*, biotinylated 50K; *lane 3*, biotinylated 50K-KGE. Positions of molecular mass markers (kDa) are shown. A band in the α5β1-Fc sample running just below the 50-kDa marker probably corresponds to a partial cleavage product of the α5 light chain-Fc subunit (α*5-Fc*).

For surface plasmon resonance (SPR) assays, we used the recombinant proteins α5β1-Fc (38), the 50-kDa fragment of fibronectin (3FN6–10, “50K”), and a control inactive mutant 50K-KGE in which the RGD sequence is converted to Lys-Gly-Glu (Fig. 1). In this assay (35), 50K is linked to the chip surface, and then recombinant α5β1-Fc is flowed over the surface for 120 s, leading to the formation of α5β1–50K complexes. Subsequently, the complexes dissociate slowly (see “Experimental Procedures” for further details). To test the ability of mAbs to affect the stability of IFCs, mAbs were injected during the dissociation phase as described previously (*i.e.* post-integrin injection) (35). Three possible outcomes would be anticipated as follows: (i) if mAbs were unable to bind to the complexes, there would be no effect on the dissociation rate; (ii) if mAbs could bind to and cause disruption of IFCs, there would be an observed increase in the dissociation rate; or (iii) if mAbs could bind to IFCs without causing disruption, there would be an increase in SPR signal due to mAb binding.

**FIGURE 2.**
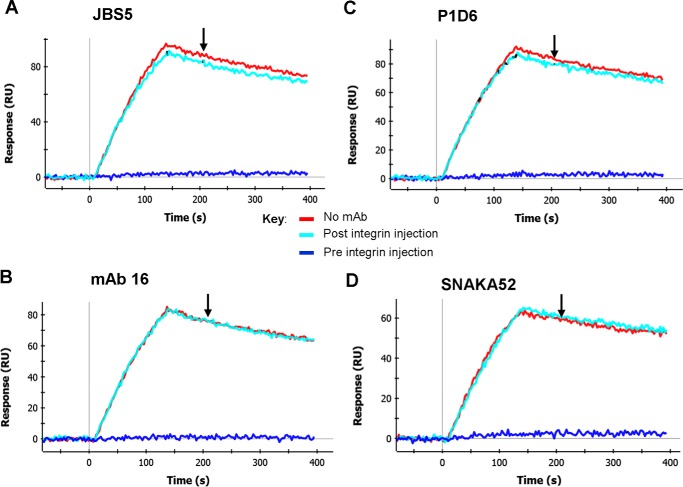
**Effect of function-blocking anti-α5 mAbs on integrin-fibronectin complexes.** *A–D*, binding of α5β1-Fc to 50K fibronectin fragment took place for 120 s in three parallel channels in RB. In the *blue sensorgrams*, integrin was pre-mixed with 100 nm of the indicated anti-α5 mAb. 60 s after the start of the dissociation phase, at the time indicated by the *downward-pointing arrow* (∼207 s), either RB alone (*red* and *blue sensorgrams*) or RB with 100 nm mAb (*cyan sensorgrams*) was injected for 120 s. *A*, dissociation rate in buffer alone was 9.73 × 10^−4^ s^−1^ and in the presence of JBS5 was 8.98 × 10^−4^ s^−1^. *B*, dissociation rate in buffer alone was 9.75 × 10^−4^ s^−1^ and in the presence of mAb was 16 9.87 × 10^−4^ s^−1^. *C*, dissociation rate in buffer alone was 9.70 × 10^−4^ s^−1^ and in the presence of P1D6 was 9.24 × 10^−4^ s^−1^. *D*, dissociation rate in buffer alone was 7.93 × 10^−4^ s^−1^ and in the presence of SNAKA52 6.44 × 10^−4^ s^−1^. Dissociation rates were measured between 208 and 330 s. Similar results were obtained in three separate experiments.

First, we tested whether the function-blocking anti-α5 subunit mAbs JBS5, SNAKA52, 16, and P1D6 were able to alter the dissociation rate of integrin-50K complexes. Key epitope residues of these mAbs lie in loop regions of the β-propeller domain (Table 1). The results (Fig. 2, *cyan sensorgrams*) showed that none of these mAbs caused an increase in the dissociation rate relative to the buffer-only channel (Fig. 2, *red sensorgrams*). As a control, when these mAbs were added to the integrin before binding to the fibronectin fragment (*i.e.* pre-integrin injection), almost complete inhibition of complex formation was observed (Fig. 2, *blue sensorgrams*). Hence, although these mAbs could prevent complexes from forming, they could not disrupt pre-formed IFCs, suggesting that these mAbs are unable to bind to the complexes.

**TABLE 1:** **The properties and epitope location of anti-α5 and anti-β1 mAbs used in this study**

mAb	Property	Epitope location
JBS5	Function blocking	α5 β-propeller domain Ser-85 (27)^1^
SNAKA52	Function blocking	α5 β-propeller domain Ser-85 (27)^1^
P1D6	Function blocking	α5 β-propeller domain Leu212 (27)
16	Function blocking	α5 β-propeller domain Glu-126/Leu-128/Trp-157 (27, 68, 69)*^a^*
VC5	Neutral	α5 β-propeller domain (70)
11	Neutral	α5 Calf-2 domain His-770/Arg-772 (71)
SNAKA51	Activating (weak)	α5 Calf-1/Calf-2 domains (39, 68)
13	Function blocking	β1 βI domain Asn-207–Lys-218 (32)
4B4	Function blocking	β1 βI domain Ly-s218 (32)
AIIB2	Function blocking	β1 βI domain Val-211 (32)
Lia1/2	Function blocking	β1 unknown
3S3	Function blocking	β1 unknown
6S6	Function blocking	β1 unknown
HUTS-4	Activating (weak)	β1 hybrid domain Ser-370/Glu-371/Lys-417 (24)
JB1A	Neutral	β1 hybrid domain Asn-82–Lys-87 (72)
8E3	Activating (weak)	β1 PSI domain Glu-4 (40)
TS2/16	Activating (strong)	β1 βI domain Asn-207–Lys-218 (32)
K20	Neutral	β1 I-EGF domains (32)

*^a^* In the original paper (27), Ser-85 was incorrectly designated as Ser-75, and Glu-126 and Leu-128 were incorrectly designated as Glu-116 and Leu-118.

##### Function-blocking Anti-β1 mAbs, with the Exception of Lia1/2, Can Disrupt Pre-formed α5β1-Fibronectin Complexes

**FIGURE 3.**
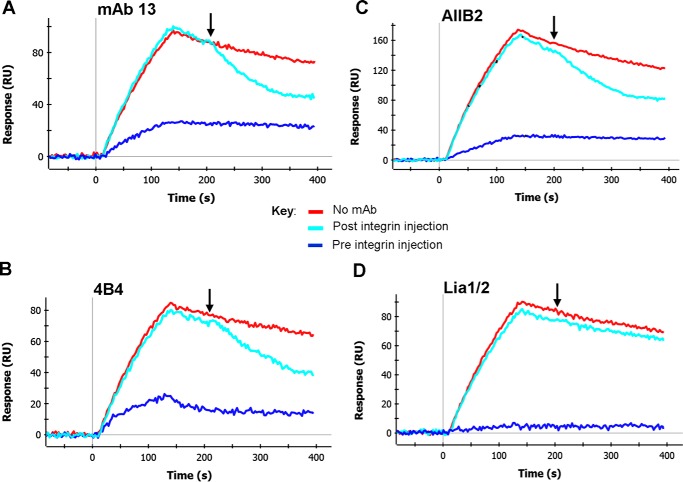
**Effect of function-blocking anti-β1 mAbs on integrin-fibronectin complexes.** *A–D*, binding of α5β1-Fc to 50K fibronectin fragment took place for 120 s in three parallel channels in RB. In the *blue sensorgrams*, integrin was pre-mixed with 100 nm of the indicated anti-β1 mAb. 60 s after the start of the dissociation phase, at the time indicated by the *downward-pointing arrow* (∼207 s), either RB alone (*red* and *blue sensorgrams*) or RB with 100 nm mAb (*cyan sensorgrams*) was injected for 120 s. Dissociation rates were measured during the buffer or mAb injection step (208–228 s). *A*, dissociation rate in buffer alone was 1.11 × 10^−3^ s^−1^ and in the presence of mAb 13 was 4.98 × 10^−3^ s^−1^. *B*, dissociation rate in buffer alone was 1.01 × 10^−3^ s^−1^ and in the presence of 4B4 was 4.04 × 10^−3^ s^−1^. *C*, dissociation rate in buffer alone was 1.37 × 10^−3^ s^−1^ and in the presence of AIIB2 was 4.31 × 10^−3^ s^−1^. *D*, dissociation rate in buffer alone was 0.91 × 10^−3^ s^−1^ and in the presence of Lia1/2 was 1.05 × 10^−3^ s^−1^. Similar results were obtained in three separate experiments.

Next, we tested whether the function-blocking anti-β1 subunit mAbs 13, 4B4, AIIB2, and Lia1/2 were able to increase the dissociation rate of IFCs. Key epitope residues of the mAbs 13, 4B4, and AIIB2 lie in the α2 helix region of the βI domain (Table 1), but the exact Lia1/2 epitope location is unknown. The results (Fig. 3, *cyan sensorgrams*) showed that 13, 4B4, and AIIB2 caused a marked (∼4-fold) increase in the dissociation rate, whereas Lia1/2 did not affect the dissociation rate (Fig. 3 legend). When these mAbs were pre-mixed with the integrin before binding to the fibronectin fragment (Fig. 3, *blue sensorgrams*), 13, 4B4, and AIIB2 showed a partial (∼70%) inhibition of complex formation, whereas Lia1/2 caused a near-complete inhibition, comparable with that seen for the function-blocking anti-α5 mAbs. Hence, 13, 4B4, and AIIB2 can bind to and disrupt IFCs; members of this group of mAbs appear to have an allosteric mode of action (35). In contrast, Lia1/2 cannot bind to or disrupt IFCs and seem to have properties similar to the anti-α5 mAbs.

##### Non-function-blocking mAbs Can Bind to but Do Not Disrupt Pre-formed α5β1-Fibronectin Complexes

**FIGURE 4.**
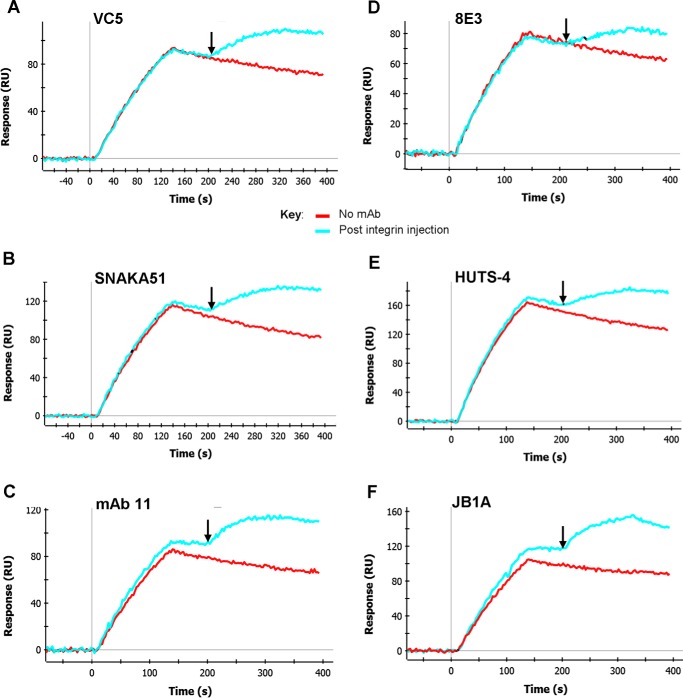
**Effect of non-function-blocking anti-α5 and anti-β1 mAbs on integrin-fibronectin complexes.** *A–F*, binding of α5β1-Fc to 50K fibronectin fragment took place for 120 s in three parallel channels in RB. In the *blue sensorgrams*, integrin was pre-mixed with 100 nm of the indicated anti-α5 mAb (*A–C*) or anti-β1 mAb (*D–F*). 60 s after the start of the dissociation phase, at the time indicated by the *downward-pointing arrow* (∼207 s), either RB alone (*red* and *blue sensorgrams*) or RB with 100 nm mAb (*cyan sensorgrams*) was injected for 120 s. Dissociation rates were measured after the end of the buffer or mAb inject step (330–390 s) for α5β1-fibronectin complexes (*red sensorgrams*) or mAb-α5β1-fibronectin complexes (*cyan sensorgrams*). *A*, dissociation rates were 8.75 × 10^−4^ and 3.59 × 10^−4^ s^−1^, respectively. *B*, dissociation rates were 11.3 × 10^−4^ and 3.26 × 10^−4^ s^−1^, respectively. *C*, dissociation rates were 8.47 × 10^−4^ and 4.47 × 10^−4^ s^−1^, respectively. *D*, dissociation rates were 7.55 × 10^−4^ and 4.33 × 10^−4^ s^−1^, respectively. *E*, dissociation rates were 8.10 × 10^−4^ and 3.28 × 10^−4^ s^−1^, respectively. *F*, dissociation rates were 6.73 × 10^−4^ and 12.9 × 10^−4^ s^−1^, respectively. Similar results were obtained in three separate experiments.

Finally, we tested the effect of the non-function-blocking anti-α5 subunit (Fig. 4, *A–C*) and anti-β1 subunit (Fig. 4, *D–F*) mAbs on α5β1-fibronectin complexes. The epitopes of these mAbs lie outside the ligand-binding region (Table 1). All of the mAbs caused an increase in SPR signal when added during the dissociation phase, indicative of mAb binding to the complexes (Fig. 4, *cyan sensograms*) without causing disruption. With the exception of JB1A, none of these antibodies caused an increase in the dissociation rate of the complexes subsequent to mAb binding (Fig. 4 legend). Furthermore, all of the other non-function-blocking mAbs were able to slightly decrease the dissociation rate, even though the measured dissociation rates of mAb-α5β1-fibronectin complexes will also include a component from the dissociation of mAb binding to the integrin. Some of these mAbs (SNAKA51, 8E3, and HUTS-4) have previously been shown to be weakly activating and hence may be capable of directly stabilizing IFCs (24, 39, 40). All the mAbs showed no inhibition of complex formation when pre-mixed with the integrin (data not shown). In summary, although all these non-function-blocking mAbs bound to IFCs, no disruption of the complexes was observed.

##### Lia1/2 Binding to α5β1 Is Strongly Perturbed by Ligand Recognition

**FIGURE 5.**
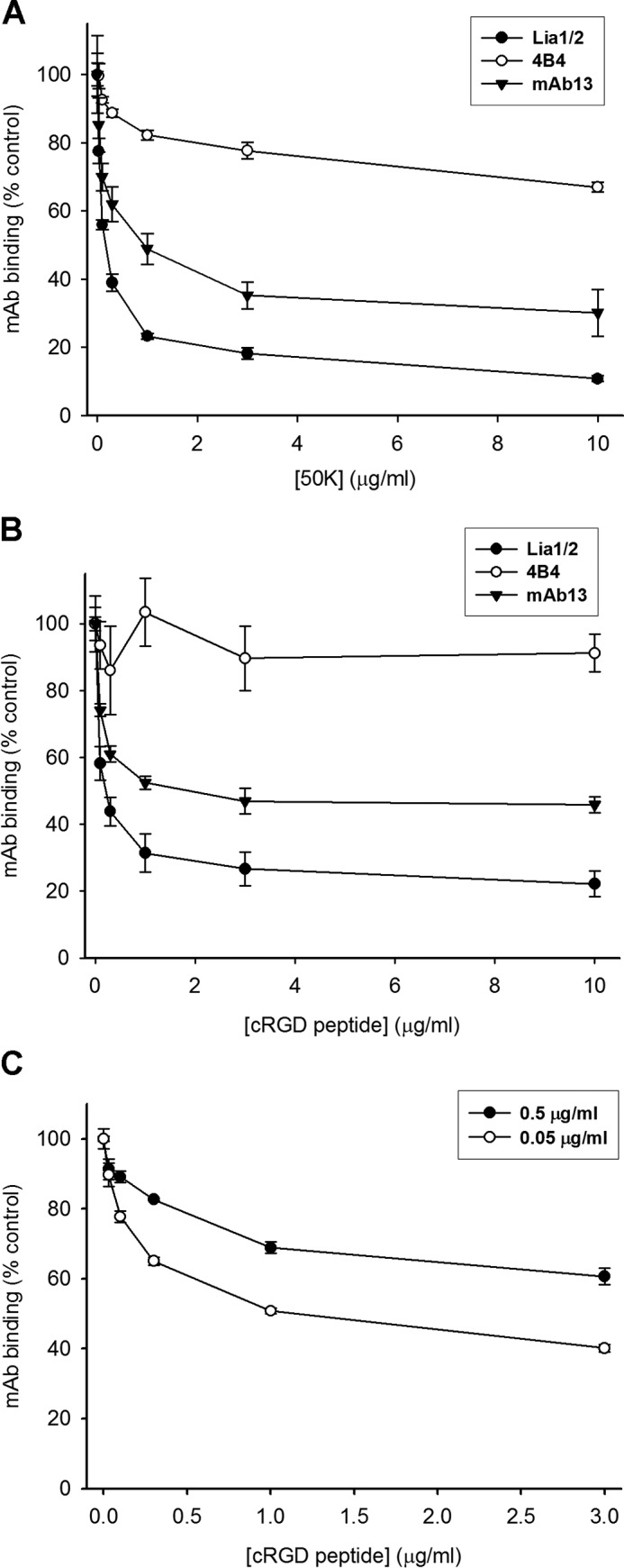
**Effect of 50K fibronectin fragment and cyclic RGD peptide on Lia1/2 binding to α5β1.** *A* and *B*, ability of 50K (*A*) or cyclic RGD peptide (*B*) to inhibit binding of Lia1/2 (*closed circles*) to α5β1 was tested in a solid-phase assay, in parallel with mAbs 13 (*closed triangles*) and 4B4 (*open circles*). *C*, test of competitive inhibition by cRGD peptide on Lia1/2 binding to α5β1. The effect of cRGD was tested at 10-fold different concentrations of Lia1/2 (0.05 and 0.5 μg/ml; *open* and *closed circles*, respectively). In this experiment, the concentration of cRGD for half-maximal inhibition of Lia1/2 binding was estimated by non-linear regression analysis to be 0.231 and 0.503 μg/ml for 0.05 and 0.5 μg/ml of Lia1/2, respectively. In *n* = 4 experiments, the concentrations of cRGD for half-maximal inhibition were 0.211 ± 0.059 and 0.538 ± 0.130 μg/ml for 0.05 and 0.5 μg/ml of Lia1/2, respectively (mean ± S.D., *p* < 0.005, Student's *t* test).

The unusual properties of Lia1/2 prompted us to explore further its mechanism of action. We previously showed that the binding of some function-blocking mAbs to β1 is inhibited by RGD-containing peptides and fibronectin fragments (15, 41). We therefore tested the effect of 50K and cRGD on the binding of Lia1/2 to α5β1, and we simultaneously compared the effect of these reagents on the binding of mAbs 13 and 4B4. The results (Fig. 5, *A* and *B*) showed that Lia1/2 binding was more strongly inhibited by 50K and cRGD than was the binding of mAbs 13 and 4B4. In control experiments (data not shown), the 50K-KGE mutant protein had no effect on mAb binding. We next tested whether the cRGD peptide acted as an allosteric or a competitive inhibitor of Lia1/2 binding (Fig. 5*C*). In this experiment, the effect of cRGD on binding of Lia1/2 to α5β1 was tested over a 10-fold range of mAb concentrations. The results (Fig. 5 legend) showed that the concentration of cRGD peptide for half-maximal inhibition increased at the higher antibody concentration. If ligand behaved as a purely allosteric inhibitor of mAb binding, the concentration of peptide for half-maximal inhibition should be unchanged, and the maximal extent of inhibition should decrease with increased antibody concentration (as we previously observed for mAb13 binding (41)). In contrast, if ligand behaved as a direct competitive inhibitor of mAb binding, the concentration of ligand for half-maximal inhibition of antibody binding should increase in proportion to the antibody concentration, and the maximal extent of inhibition should be unchanged. However, for Lia1/2 binding, we observed that the concentration of cRGD peptide for half-maximal inhibition increased 2–3-fold for a 10-fold increase in antibody concentration, and the maximal extent of inhibition decreased. These data indicate that the inhibition by cRGD is partly competitive and partly allosteric in nature.

##### Epitope Mapping of Lia1/2

The epitope of Lia1/2 has not been accurately mapped. We initially used mouse/human and chicken/human β1 chimeras to localize the epitope to a specific region of β1. The results (Table 2) showed that the Lia1/2 epitope was included in a region containing amino acids 190–304, which is part of the βI domain. The same region has previously been shown to include the epitopes of mAbs 13, 4B4, AIIB2, and TS2/16 (32). Because there are only five amino acid changes in this region from mouse to human β1, point mutations in each of these five residues (Asn-207, Lys-208, Val-211, Lys-218, and Met-287) were made, essentially as described previously (32). The results (Tables 3 and 4) showed that no single point mutation abrogated Lia1/2 binding, although this is also the case with several other mAbs that bind to this region, including 13 and TS2/16 (32). Four of the mutated residues, Asn-207, Lys-208, Val-211, and Lys-218, lie within the α2 helix region of the βI domain (17, 18). However, Lia1/2 did react with the previously described “CH mutant” (32), which contains the sequence of human β1 residues 207–218 in the context of chicken β1 (data not shown), and hence its epitope directly overlaps with that of other function-perturbing anti-β1 mAbs.

**TABLE 2:** **The reactivity of Lia1/2 to wild-type and different chimeric β1 chains by flow cytometry** The numbers in the table represent mean fluorescence intensity values in the typical experiments using CHO cells stably expressing wild-type or chimeric β1. /+ represents positive reactivity of the transfected cells to the mAbs; /− represents no reactivity. Mouse IgG was used as a control.

Antibody	CHO	h587/m	h425/m	h354/m	h304/c	h189/c
Mouse IgG	3.52	4.48	4.05	4.92	6.24	5.49
Lia1/2	4.33/−	75.01/+	40.26/+	163.51/+	71.45/+	5.22/−

**TABLE 3:** **Reactivity of Lia1/2 to wild-type and mutant human β1 chains by flow cytometry** Human β1 chains with human-to-mouse mutations were transiently expressed on CHO cells, and the reactivity of the cells to mAbs was examined by flow cytometry; numbers represent mean fluorescence intensity values. Clonal CHO cells stably expressing human wild-type β1 were used as positive control. /+ represents positive reactivity to the mAbs.

Antibody	CHO	N207D	K208R	V211F	K218Q	Human β1
Mouse IgG	3.93	2.73	4.47	2.99	5.08	3.92
Lia1/2	4.61/−	183.79/+	126.30/+	108.92/+	54.86/+	260.94/+

**TABLE 4:** **Reactivity of Lia1/2 to wild-type and mutant human β1 chains by flow cytometry** CHO-K1 cells transiently expressing wild-type or M287V mutant β1-GFP were examined by flow cytometry for reactivity with TS2/16 or Lia1/2; numbers represent MFI values. /+ represents positive reactivity to the mAbs. Mouse IgG was used as a control.

Antibody	M287V	Human β1
Mouse IgG	247	270
TS2/16	11,512/+	11,780/+
Lia1/2	14,856/+	14,111/+

##### Anti-β1 mAbs That Induce Homotypic Aggregation Fail to Disrupt α5β1-Fibronectin Complexes

**FIGURE 6.**
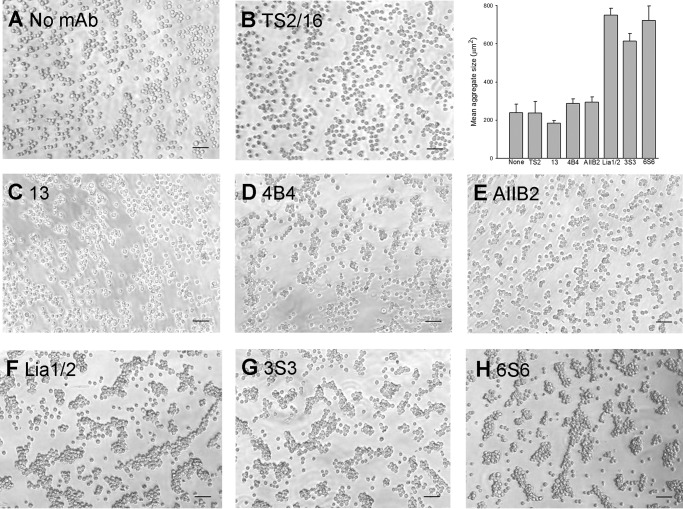
**Effect of anti-β1 mAbs on homotypic aggregation of Jurkat cells.** *A–H*, cells (5 × 10^5^/ml) were incubated for 2 h at 37 °C in the presence of the indicated mAbs and photographed using a phase-contrast microscope. *Scale bars,* 50 μm. *Inset* shows *histogram* of mean aggregate size, averaged over three fields of view (mean ± S.D.) for no mAb (*None*), TS2/16 (*TS2*), 13, 4B4, AIIB2, Lia1/2, 3S3, and 6S6. The experiment shown is representative of three separate experiments.

Treatment of mononuclear cells with some function-blocking anti-β1 mAbs can cause homotypic cell aggregation (42–47), a poorly understood phenomenon that requires integrin-mediated intracellular signaling (42, 48). The epitopes of many function-blocking anti-β1 mAbs have previously been sub-divided into two sub-classes, A1 and A2; although the epitopes of these two classes overlap, members of the A1 class can be distinguished from those of the A2 class by their ability to induce homotypic aggregation (44). The A1 class is known to include Lia1/2 (44). We tested the ability of the function-blocking anti-β1 mAbs used in this study to induce homotypic aggregation of Jurkat T-lymphoblastic cells, using the activating mAb TS2/16 as a negative control (Fig. 6, *A–F*). Our results showed that mAb 13 did not cause cell aggregation, and 4B4 and AIIB2 caused only the appearance of some small aggregates of cells. In contrast, Lia1/2 caused the appearance of very large clusters of cells, and so was the only mAb of this group able to strongly trigger homotypic aggregation. Hence, mAbs 13, 4B4, and AIIB2 are members of the A2 class.

**FIGURE 7.**
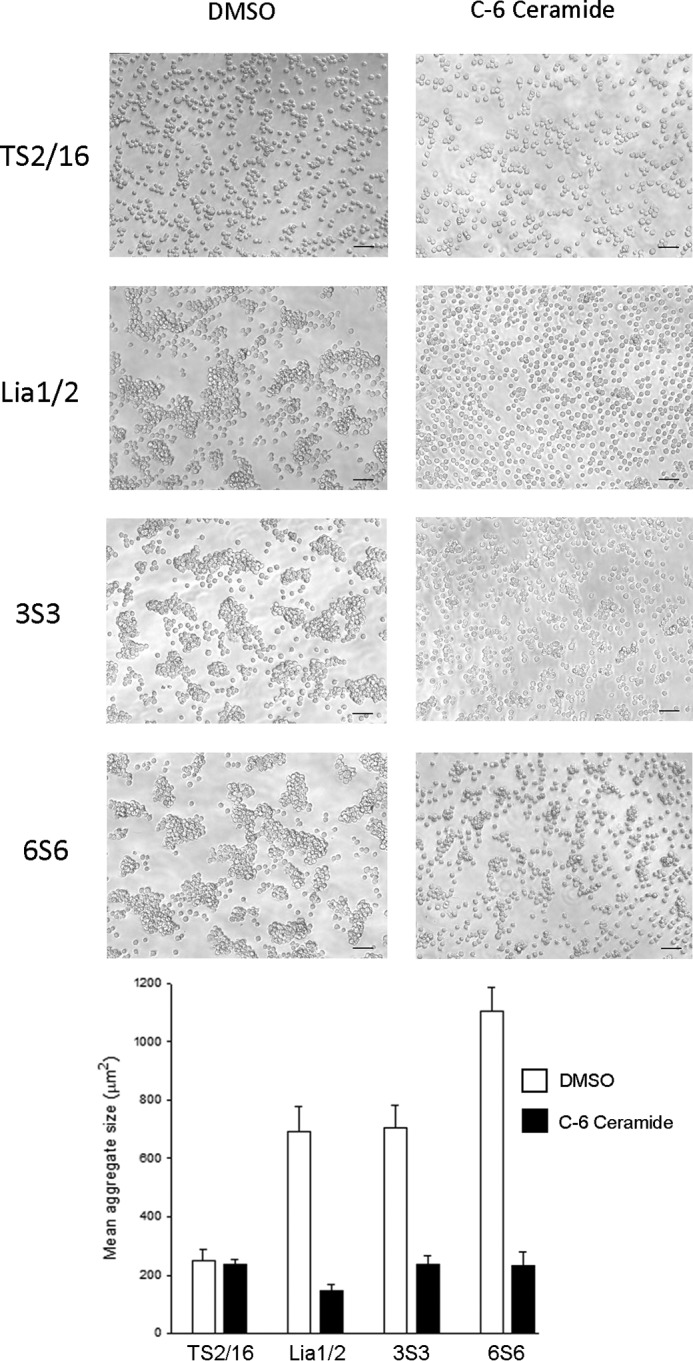
**Effect of C_6_-ceramide on homotypic cell aggregation stimulated by anti-β1 mAbs.** Jurkat cells (8 × 10^5^/ml) were incubated for 2 h at 37 °C in the presence of the indicated mAbs, in the presence of DMSO (solvent control) or 10 μm C_6_-ceramide, and photographed using a phase-contrast microscope. C_6_-ceramide did not cause any reduction in cell viability over the time course of the experiment (>97% of cells were viable both before and after the experiment, as determined by propidium iodide staining, data not shown). *Scale bars,* 50 μm. TS2/16 is used as a negative control mAb in this assay. *Inset* shows *histogram* of mean aggregate size, averaged over three fields of view (mean ± S.D.). The experiment shown is representative of three separate experiments.

We next tested whether other anti-β1 mAbs that have been reported to induce homotypic aggregation had properties similar to those of Lia1/2. We obtained the mAbs 3S3 and 6S6 (47) and confirmed that these mAbs were able to strongly induce cell aggregation (Fig. 6, *G* and *H*). It has been previously shown that cell aggregation induced by some anti-β1 mAbs can be specifically inhibited by ceramide analogs (42). Aggregation caused by Lia1/2, 3S3, and 6S6 was blocked to a similar extent by the cell-permeable compound *N*-hexanoyl-d-sphingosine (C_6_-ceramide) (Fig. 7), indicating that all three mAbs trigger cell aggregation via a common mechanism involving intracellular signaling.

**FIGURE 8.**
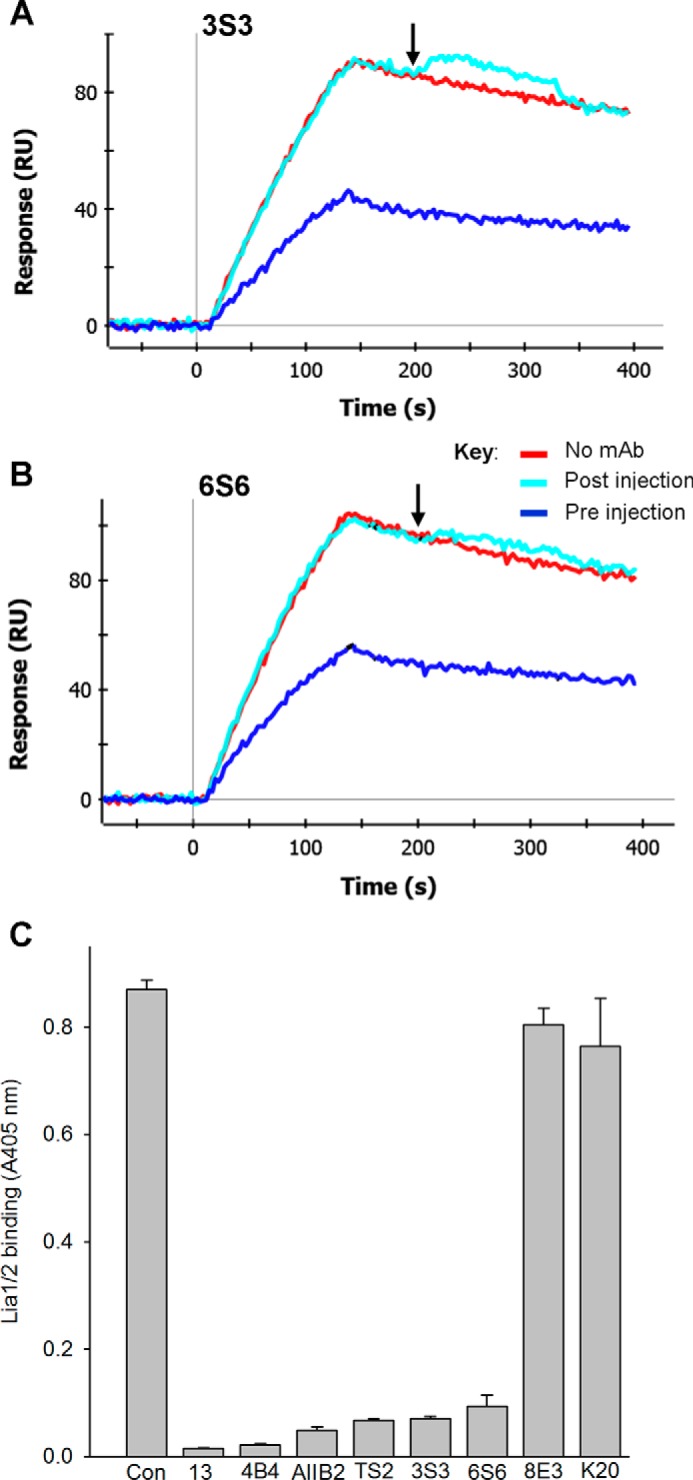
**Characterization of mAbs 3S3 and 6S6.** *A* and *B*, SPR test of competitive or allosteric inhibition by 3S3 and 6S6. Binding of α5β1-Fc to 50K fibronectin fragment took place for 120 s in three parallel channels in RB. In the *blue sensorgrams*, integrin was pre-mixed with 200 nm 3S3 (*A*) or 6S6 (*B*). 60 s after the start of the dissociation phase, at the time indicated by the *downward-pointing arrow* (∼207 s), either RB alone (*red* and *blue sensorgrams*) or RB with 200 nm mAb (*cyan sensorgrams*) was injected for 120 s. *C*, competitive ELISA experiment to test whether epitopes of 3S3 and 6S6 overlap with that of Lia1/2. Binding of biotinylated Lia1/2 to α5β1-Fc was competed by a large excess of unlabeled mAbs 13, 4B4, AIIB2, 4B4, TS2/16 (*TS2*), 3S3, 6S6, 8E3, or K20. *Con*, control. Similar results were obtained in three separate experiments.

We then examined the effect of 3S3 and 6S6 on the formation and dissociation of α5β1-fibronectin complexes (Fig. 8, *A* and *B*). The two mAbs inhibited the formation of IFCs (Fig. 8, *A* and *B*, *blue sensorgrams*), although they were less potent inhibitors of complex formation than Lia1/2. Significantly, like Lia1/2, both 3S3 and 6S6 were unable to disrupt pre-formed IFCs (Fig. 8, *A* and *B*, *cyan sensorgrams*). A very small increase in signal was consistently observed during the time window that these mAbs were injected over the complexes, suggesting that they could bind very weakly to the complexes, although this binding had no effect on the overall dissociation rate.

The epitopes of 3S3 and 6S6 have not been localized to a specific region of β1. To test whether the epitopes of these mAbs map to the same region of β1 as Lia1/2, we used a competitive ELISA (Fig. 8*C*). In this assay, we used biotinylated Lia1/2 and competed its binding to α5β1 using a large excess of unlabeled mAbs. The binding of biotinylated Lia1/2 was abrogated by the mAbs 13, 4B4, and AIIB2, as well as TS2/16 (32), in agreement with our epitope mapping data above. Significantly, both 3S3 and 6S6 also strongly inhibited Lia1/2 binding, whereas the control mAbs 8E3 and K20, whose epitopes lie outside of the βI domain (Table 1), had little effect on Lia1/2 binding. Therefore, the epitopes of 3S3 and 6S6 appear to be closely overlapping with the epitope of Lia1/2. Hence, like Lia1/2, 3S3 and 6S6 are members of the A1 class.

##### New Model of the α5β1-Fibronectin Complex Supports the Epitope-masking Hypothesis

**FIGURE 9.**
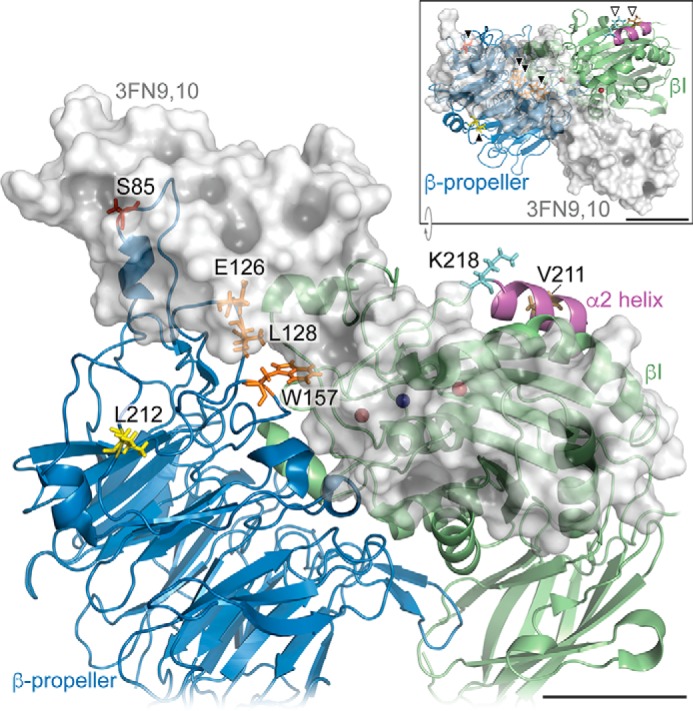
**Model of headpiece region of α5β1 bound to 3FN9,10, showing epitopes of function-blocking mAbs.** In the model, the α5 subunit is shown as a *blue ribbon*, β1 subunit as a *green ribbon*, and 3FN9,10 as a *semi-transparent light gray surface*. In the α5 subunit, residue Leu-212 (P1D6 epitope) is shown in *yellow*; residues Glu-126, Leu-128, and Trp-157 (mAb 16 epitope) are shown in *orange*, and residue Ser-85 (JBS5 and SNAKA52 epitopes) is shown in *red*. In the β1 subunit, residues Val-211 (AIIB2 epitope) and Lys-218 (4B4 epitope) are shown in *brown* and *cyan*, respectively. The MIDAS Mg^2+^ ion is depicted as a *dark blue sphere*; Ca^2+^ ions at the SyMBS (synergistic metal-binding site) and ADMIDAS (adjacent to MIDAS) sites (flanking the MIDAS site) are shown as *dark red spheres*. The α2 helix in the βI domain is shown in *magenta*. Residues that contribute to the epitopes of mAbs 13, 4B4, and AIIB2 lie in the α2 helix (5). *Inset* shows an *x* axis-rotated view, with residues labeled in the main model indicated by *arrowheads* for reference (α5 subunit residues, *black arrowheads*; β1 subunit residues, *white arrowheads*). *Scale bars,* 20 Å.

**FIGURE 10.**
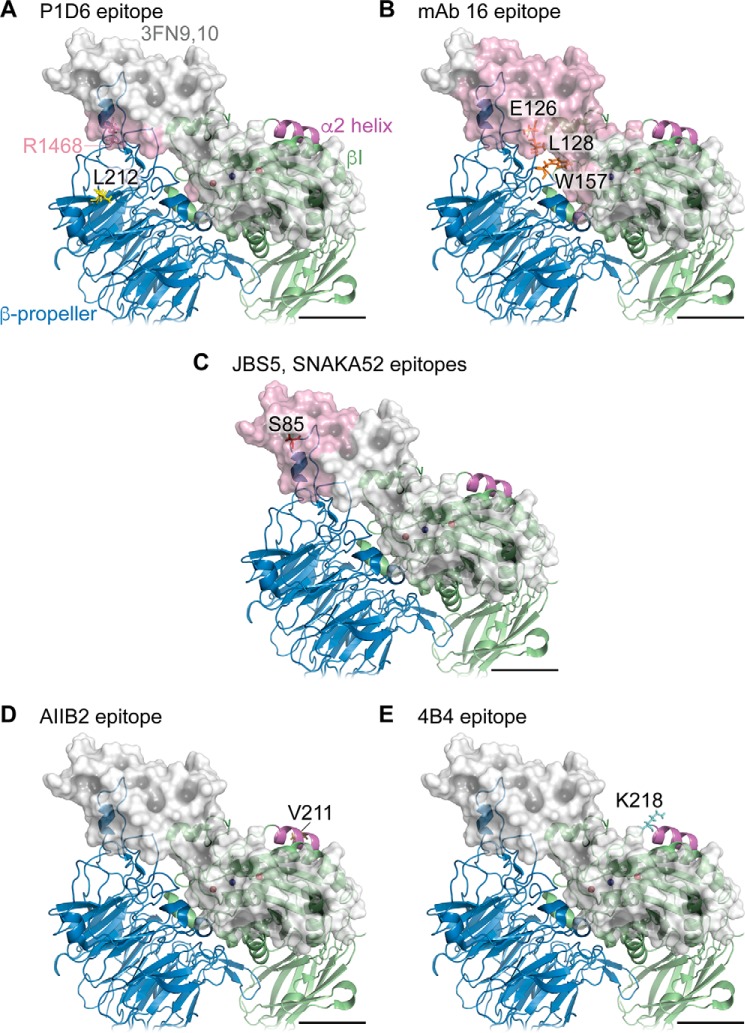
**Putative accessibility of epitopes of function-blocking mAbs in a model of the headpiece of α5β1 bound to 3FN9,10.** *A–E*, molecular model is the same as that shown in Fig. 9. In respective panels, residue Leu-212 (P1D6 epitope) is shown in *yellow*; residues Glu-126, Leu-128, and Trp-157 (mAb 16 epitope) are shown in *orange*; residue Ser-85 (JBS5 and SNAKA52 epitopes) is shown in *red* within the α5 subunit (*blue ribbon*); residue Val-211 (AIIB2 epitope) is shown in *brown*, and residue Lys-218 (4B4 epitope) is shown in *cyan* within the β1 subunit (*green ribbon*). The α2 helix in the βI domain is shown in *magenta*. 3FN9,10 is shown as a *semi-transparent light gray surface*, and the surface of accessible 3FN9,10 residues within 20 Å of indicated mAb epitope residues is rendered in *semi-transparent pink*. Residue Arg-1468 (from the Pro-Pro-Ser-Arg-Asn synergy sequence of murine fibronectin) is shown in *dark pink* and indicated by the *pink text* in *A*. Anti-α5 mAbs would be sterically hindered from binding to indicated epitope residues by fibronectin, whereas anti-β1 mAbs would not. *Scale bars,* 20 Å.

The failure of some function-blocking mAbs to disrupt IFCs suggests that these mAbs are unable to bind to IFCs because their epitopes become masked in the ligand-bound state of α5β1. To test this possibility further, a model of the α5β1-fibronectin complex (Fig. 9 and supplemental Video S1) was constructed based on the crystal structure of 3FN10 bound to αVβ3 (49) and the NMR structure of murine 3FN9,10 (50). 3FN9 contains the synergy region, whereas 3FN10 contains the RGD sequence. The orientation of 3FN9,10 was guided by docking the fragment onto the α5β1 crystal structure (18), with 3FN10 in the same orientation as in the αVβ3 crystal structure (49). Only minimal adjustments in the position of 3FN9 were required to prevent steric clashes between 3FN9 and β-propeller residues (see “Experimental Procedures”). To estimate the antibody-accessible surface, we rolled a 20-Å sphere (the approximate size of a Fab fragment) over the structure (51). The results (Fig. 10) indicated that α5 residues Leu-212 (P1D6 epitope), Glu-126/Leu-128/Trp-157 (mAb 16 epitope), and Ser-85 (JBS5 and SNAKA52 epitopes) would not be accessible in the ligand-occupied state. In contrast, β1 residues Val-211 and Lys-218, forming part of the AIIB2 and 4B4 epitopes, respectively, would be accessible. The epitopes of non-function-blocking mAbs (data not shown) were also fully available in this model. The exact location of the Lia1/2 epitope is not known, but it appears to lie closer to 3FN10 than the epitopes of AIIB2 and 4B4.

##### Only Allosteric Anti-β1 mAbs Can Cause Complete Detachment of Fibrosarcoma Cells Pre-spread on Fibronectin

**FIGURE 11.**
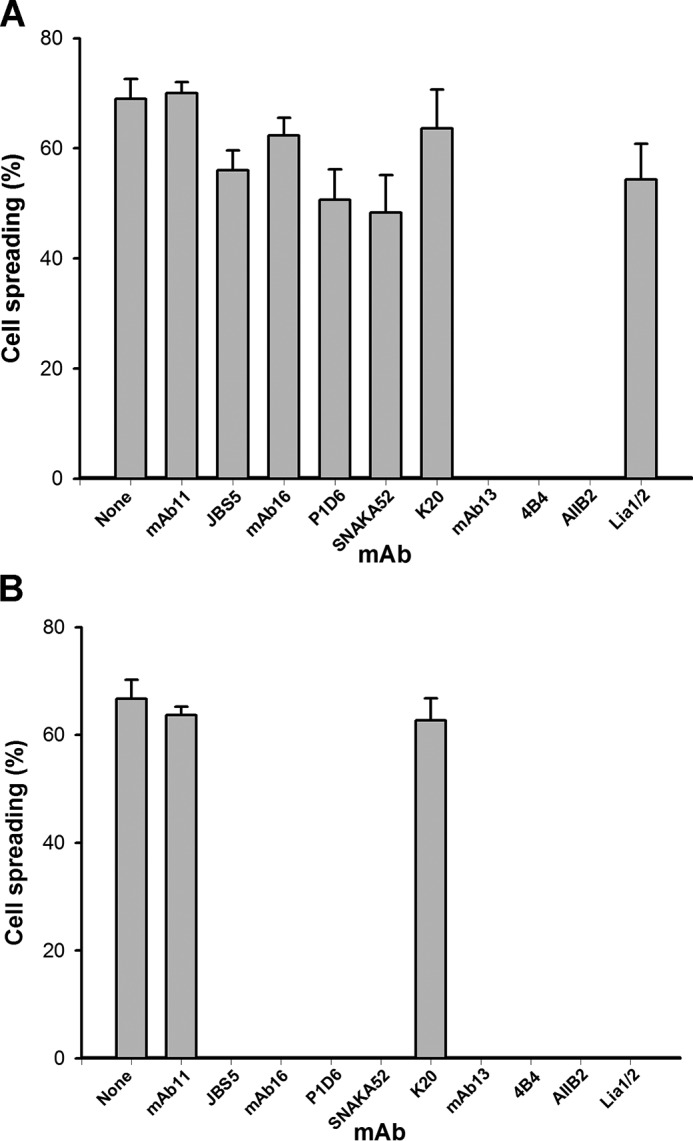
*A,* effect of function-blocking mAbs on HT-1080 cells pre-spread on 50K. Cells were allowed to spread on 50K for 1 h and then exposed to the indicated anti-α5 (JBS5, 16, P1D6, or SNAKA52) or anti-β1 (13, 4B4, AIIB2, or Lia1/2) mAbs for 1 h. The percentage of spread cells was then quantitated. The non-function-blocking anti-α5 mAb 11 and anti-β1 mAb K20 were used as controls. *B*, effect of the same mAbs in an assay in which the mAbs were added to the cells before spreading on 50K. Similar results were obtained in three separate experiments.

Finally, to examine the relevance of our findings to cell-surface α5β1, we tested the ability of function-blocking anti-α5 and anti-β1 mAbs to cause rounding up of HT-1080 cells pre-spread on 50K (*i.e.* with cell-surface α5β1 in complex with ligand). The results (Fig. 11*A*) showed that the allosteric anti-β1 mAbs 13, AIIB2, and 4B4 could cause complete rounding up of cells (no spread cells remaining). In contrast, the function-blocking anti-α5 mAbs JBS5, 16, P1D6, and SNAKA52, and the anti-β1 mAb Lia1/2 caused only a slight reduction (typically < 25%) in the degree of cell spreading. In control experiments, run in parallel, Lia1/2 and the function-blocking anti-α5 mAbs were potent inhibitors of cell spreading when added to the cells before plating on 50K (Fig. 11*B*).

## Discussion

The major findings of this report are as follows. (i) Function-blocking anti-α5 mAbs cannot cause dissociation of IFCs, and the epitopes of these mAbs appear to be masked in the ligand-occupied state. (ii) Allosteric function-blocking anti-β1 mAbs (A2 class) can cause disruption of IFCs, and the epitopes of these mAbs do not appear to be masked by ligand. (iii) Members of a second group of function-blocking anti-β1 mAbs (A1 class), which includes Lia1/2, fail to disrupt IFCs, suggesting that their epitopes are obscured in the IFC. (iv) In accord with our *in vitro* data, only allosteric mAbs could disrupt cell-surface α5β1-fibronectin interactions.

A preceding study defined the A1 and A2 classes of inhibitory anti-β1 mAbs (44). From our results, it appears that the members of the A1 class are distinguished from the A2 class not only by their ability to induce cell aggregation but also by their inability to dissociate IFCs. Lia1/2 appears to have a similar mode of inhibition to the function-blocking anti-α5 mAbs, acting in a predominantly steric, rather than allosteric, manner. The partially competitive effect of RGD peptide on Lia1/2 binding supports the notion that the Lia1/2 epitope may lie closer to the binding site of 3FN10 than the epitopes of other function-blocking anti-β1 mAbs, leading to masking of its epitope in the IFC.

The mechanism of anti-β1 mAb-induced cell-cell aggregation remains unclear, although previous studies (43–46) have demonstrated that this aggregation requires α4β1 integrins. There is evidence that these mAbs induce a functional association between β1 and CD98 and CD147 and that this complex causes intracellular signals that result in the induction of cell-cell adhesiveness (48). Similar to a previous report with the anti-β1 mAb MEM 101A (42), C_6_-ceramide blocked the aggregation of Jurkat cells promoted by Lia1/2, 3S3, and 6S6, suggesting the involvement of similar intracellular signaling pathways by all these mAbs. Recent studies with tumor cells suggest that these pathways involve members of the PKC family (52). Elucidation of the precise mechanisms by which A1 class mAbs cause different signals to those of the A2 class will require further investigation. However, the distinct (but overlapping) epitope location of these two classes provides a novel insight into this conundrum. Aggregation of T-cells is important *in vivo* for T-cell maturation (53); it is therefore intriguing to speculate that A1 class mAbs mimic the function of one or more natural proteins that can bind to this region of the β1 subunit.

An important inference of our studies is that function-blocking anti-integrin subunit mAbs are not all equivalent (as has often been assumed). It is important for researchers to test several different mAbs to find the one that functions best in their specific application. Our findings demonstrate that, particularly in assays where cells or purified integrins are pre-bound to the ligand, the choice of mAb is critical. Only allosteric mAbs have the capacity to disrupt pre-existing interactions.

It could be argued that the inability of some function-blocking mAbs to perturb pre-formed cell-surface integrin-fibronectin interactions (Fig. 11*A*) was due to poor accessibility of these mAbs to cell adhesion sites. We consider this explanation to be unlikely because the allosteric mAbs cause complete disruption of cell spreading, and hence these mAbs must be able to access the adhesion sites. Instead, the failure of certain mAbs to markedly disrupt cell spreading appears to correlate with our *in vitro* data showing that the epitopes of these mAbs are occluded in the IFC. A slow turnover of cell adhesion sites (which would re-expose integrin epitopes) may account for the small amount of inhibition of cell spreading by the non-allosteric inhibitory mAbs seen over the 1-h time period of the assay.

In our new model of the IFC (Figs. 9 and 10), the synergy region of 3FN9 lies very close to the residues Trp-208 and Ile-210 in the α5 subunit β-propeller domain, as proposed previously (15, 54). Significantly, P1D6 (whose epitope includes Leu-212) only blocks interaction of α5β1 with fibronectin fragments that contain the synergy region, suggesting that it binds close to a site on the α5 subunit that interacts with the synergy region of 3FN9 (15). Nevertheless, there has been some dispute as to whether or not the synergy region binds directly to the α5 subunit. For example, a lack of interaction of 3FN9 with the α5 subunit β-propeller domain was observed by negative staining and electron microscopy (55). In contrast, mutagenesis of α5 residues close to Leu-212, including Trp-208 and Ile-210, or the equivalent Trp-204 and Ile-206 in zebrafish α5, specifically blocks interaction of α5β1 with fibronectin fragments that contain the synergy region (15, 54). In addition, the W204N mutation leads to loss of α5β1 function in zebrafish development (56). Because the interaction of 3FN9 with the α5 subunit is weak, it could easily be disrupted by the high ionic strength conditions used for negative staining. Our new data support a direct interaction of 3FN9 with the Leu-212 region of the β-propeller because they provide evidence that the epitope of P1D6 is masked in the ligand-occupied state, and so, like the other α5 subunit mAbs, the epitope of P1D6 lies close to an interface between 3FN9 and the β-propeller. Binding of P1D6 to the α5 subunit is strongly inhibited by 3FN9-containing fragments (15), consistent with our model. An alternative model of the complex between 3FN9,10 and α5β1 has been previously proposed by Nagae *et al.* (17), in which 3FN9,10 lies in a groove between the α and β subunits. Our model uses the same 3FN9,10 structure as the Nagae model but incorporates new information on the position of 3FN10 (49) to direct the orientation of 3FN9. In the Nagae model, although the epitope residues of mAbs JBS5, SNAKA52, and 16 would still be predicted to be obscured by fibronectin binding, the P1D6 epitope residue Leu-212 would be minimally masked. The principle of epitope occlusion used here could also be utilized in other receptor-ligand systems to help define the boundary of the receptor-ligand interface.

A recent study of the location of epitopes in integrin α subunits identified the 4-1 loop of β-propeller blade 2 (which in α5 contains the JBS5 and SNAKA52 epitopes) and the 3-4 loop of β-propeller blade 3 (P1D6 epitope in α5) as the site of >80% of function-blocking mAb epitopes (29). Hence, the results of our study are likely to be applicable to a large proportion of other α subunit mAbs, *i.e.* epitope masking by ligand occupancy is likely to be a common feature in other integrins.

Accordingly, it is important to note that the epitopes of some therapeutic anti-integrin mAbs, such as the anti-α4 mAb Tysabri/natalizumab and the anti-αV mAb 17E6, may be partly obscured in the ligand-occupied state (30, 31). Consequently, complete blockade of integrin function by these biologicals would be dependent on turnover of integrin-ligand complexes to re-expose integrin epitopes, and this turnover can be very slow (35, 57–60). For example, we previously observed that α5β1-fibronectin complexes gradually transition to a very long-lived state, *k*_off_ ∼10^−5^ s^−1^, *i.e. t*_½_ ∼19 h (35). Our study suggests that epitope masking could reduce the effectiveness of drugs like Tysabri, and in the future it will be important to test whether their efficacy could be enhanced by co-administration of an allosteric inhibitor. Epitope masking could also have contributed to the lack of efficacy of some blocking anti-integrin mAbs in clinical trials (7–9).

In summary, our findings re-emphasize the importance of allosteric inhibition for effective blockade of integrin function (35). Allosteric anti-β1 mAbs such as AIIB2 are already showing therapeutic potential for radiosensitization of tumors and prevention of metastasis (61–63). Finally, ligand-induced epitope masking may occur in other receptor-ligand systems, and in the future it will be important to address whether this phenomenon limits the efficacy of non-integrin function-blocking therapeutic mAbs.

## Experimental Procedures

### 

#### 

##### Materials

The production of recombinant integrin α5β1-Fc has been previously described (38). In brief, α5β1-Fc was produced in NS0 cells and purified from culture medium using protein A-Sepharose. mAb TS2/16 was a gift from F. Sánchez-Madrid (Hospital de la Princesa, Madrid, Spain); mAbs JB1A, 3S3, and 6S6 were gifts from J. A. Wilkins (University of Manitoba, Winnipeg, Manitoba, Canada); and mAbs 11, 13, and 16 were gifts from K. M. Yamada (National Institutes of Health, Bethesda). 4B4 was from Beckman Coulter (UK). SNAKA51, 8E3, and SNAKA52 have been previously described (39–40, 64). P1D6, AIIB2, and HUTS-4 were from Merck Millipore (UK). VC5 and JBS5 were purchased from Pharmingen and Serotec (UK), respectively. K20 was a gift from P. T. Caswell (University of Manchester, Manchester, UK), and Lia1/2 was from Biorbyt (UK). For competitive ELISAs, Lia1/2 was biotinylated using sulfo-*N*-hydroxysuccinimide-LC-biotin (64). Further details of all the mAbs used in this study are shown in Table 1.

The 50-kDa cell-binding domain fragment of human fibronectin (3FN6–10, 50K) and the inactive mutant (50K-KGE) were produced in *Escherichia coli* and purified as before (15, 16). 50K and 50K-KGE were biotinylated and purified by gel filtration as described previously (35). Both proteins contain a single site for biotinylation in 3FN7. Avidin (from egg white) was obtained from Life Technologies, Inc. Peptide GCRGDSPCG (cRGD) was purchased from Peptide 2.0, Inc., and cyclized by oxidation as described previously (16). *N*-Hexanoyl-d-sphingosine (C_6_-ceramide), ExtrAvidin peroxidase, bovine serum albumin (BSA), and mouse IgG were purchased from Sigma.

##### SPR

Experiments were performed using the ProteOn XPR36 instrument (Bio-Rad). Running buffer (RB) was 150 mm NaCl, 10 mm HEPES, 0.05% (w/v) Tween 20, pH 7.4, with 1 mm MnCl_2_. Immobilization of avidin was performed on a GLC chip (Bio-Rad) in the vertical orientation. Two channels were activated with 150 μl of a 1:1 mixture of 20 mm *N*-ethyl-*N*′-(3-dimethylaminopropyl)carbodiimide and 11 mm sulfo-*N*-hydroxysuccinimide in water at a flow rate of 30 μl/min. Avidin was diluted in RB to a final concentration of 1.2 μm, and 150 μl was injected, followed by an injection of 150 μl of 1 m ethanolamine-HCl, pH 8.5, at a flow rate of 30 μl/min. The immobilization level of avidin was ∼3200 resonance units. Next, 150 μl of biotinylated 50K-KGE in one vertical channel and biotinylated 50K in the second (20–200 nm) in RB were injected to allow their capture by the immobilized avidin. Injection of 50K and 50K-KGE was repeated as necessary to give equivalent immobilization levels of the two proteins. The 50K-KGE channel was used as a reference. Final immobilization levels were in the range 100–300 resonance units. All experiments were performed at 25 °C.

To test the effect of mAbs on the dissociation rate of integrin-50K complexes, 100 μl of α5β1-Fc in RB was injected in three parallel horizontal channels at 50 μl/min, and binding to 50K was allowed to occur for 120 s, followed by injection of RB for 60 s. Next, either RB alone (channel 1) or RB with 100 nm mAb (channel 2) was injected for 120 s, followed by a return to RB alone. In channel 3, α5β1-Fc was pre-mixed with 100 nm mAb before injection onto the chip. The three-stage injection described above (integrin-buffer-buffer or integrin-buffer-mAb) was accomplished using the “Coinject Analyte” command. Injection quality was set at “maximum.” The integrin concentration used was 6 nm. In experiments with 3S3 and 6S6, the concentration of mAb was 200 nm and integrin concentration was 3 nm. The concentrations of mAb used gave a maximal effect in the assays.

All binding sensorgrams were collected, processed, and analyzed using the integrated ProteOn Manager software (Bio-Rad). Short black segments on some sensorgrams represent artifact (spike) removal from the data. Dissociation rates (*k*_off_) were calculated using off-rate analysis (ProteOn Manager software) using Equation 1,

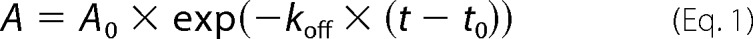
 where *A*_0_ = binding at time *t*_0_. All results shown are representative of at least three separate experiments.

##### Epitope Mapping of Lia1/2

Mapping of the Lia1/2 epitope was performed essentially as described previously for other function-perturbing anti-β1 mAbs (32, 41). The M287V human-to-mouse mutation was made in human β1-GFP (65) using the QuickChange XL mutagenesis kit (Agilent Technologies) according to the manufacturer's instructions. CHO-K1 cells were transiently transfected with either wild-type or mutant integrin by nucleofection (Lonza), and the binding of Lia1/2 was detected by flow cytometry using an LSR Fortessa cytometer (BD Biosciences) with AlexaFluor 647 anti-mouse IgG (Stratech) as secondary antibody after gating the cells for those expressing GFP. These assays were repeated twice, with the same overall results.

##### Effect of 50K and cRGD on mAb Binding

Purified α5β1-Fc was diluted 1:1000 with Dulbecco's PBS (final concentration ∼2 μg/ml), and 50-μl aliquots were added to the wells of a 96-well ELISA plate (Costar ½-volume). Plates were incubated overnight at room temperature, and wells were blocked for 1–3 h with 200 μl of 5% (w/v) BSA, 150 mm NaCl, 0.05% (w/v) NaN_3_, 25 mm Tris-HCl, pH 7.4. Wells were then washed three times with 200 μl of 150 mm NaCl, 25 mm Tris-HCl, pH 7.4, containing 1 mg/ml BSA (buffer A) with 1 mm MnCl_2_. mAbs (0.1 μg/ml) in buffer A with 1 mm MnCl_2_ were added to the wells in the presence of varying concentrations of cRGD or 50K. The plate was then incubated at room temperature for 2 h. Unbound antibody was aspirated, and the wells were washed three times with buffer A. Bound antibody was quantified by addition of 1:1000 dilution of goat anti-mouse or anti-rat peroxidase conjugate (Jackson ImmunoResearch) in buffer A with 1 mm MnCl_2_ for 30 min at room temperature. Wells were then washed four times with buffer A, and color was developed using 2,2′-azino-bis(3-ethylbenzothiazoline-6-sulfonic acid) substrate. Measurements obtained were the means ± S.D. of four replicate wells. Experiments examining the effect of varying concentrations of cRGD on the binding of Lia1/2 at 0.05 and 0.5 μg/ml were performed in the same manner.

##### Competitive ELISA

ELISA plates were coated with α5β1-Fc, blocked, and washed as described above. Biotinylated Lia1/2 (0.1 μg/ml) in buffer A was added to the wells in the absence or presence of a large excess of unlabeled competitor mAbs (10 or 30 μg/ml for 3S3 and 6S6). The plate was then incubated at room temperature for 2 h. Unbound antibodies were aspirated, and the wells were washed three times with buffer A. Bound biotinylated antibody was quantified by the addition of 1:500 dilution of ExtrAvidin peroxidase conjugate in buffer A for 30 min at room temperature. Wells were then washed four times with buffer A, and color was developed using 2,2′-azino-bis(3-ethylbenzothiazoline-6-sulfonic acid) substrate. Measurements obtained were the means ± S.D. of four replicate wells. In all the plate assays described above, the amount of nonspecific binding was measured by determining the level of antibody binding to wells coated with BSA alone; these values were subtracted from the corresponding values for receptor-coated wells. Each experiment shown is representative of at least three separate experiments.

##### Cell Aggregation Assays

Jurkat cells were maintained in complete RPMI 1640 medium (supplemented with 2 mm glutamine and 10% fetal bovine serum). Aggregation assays were performed essentially as described by Lee *et al.* (42). Cells (5 × 10^5^/ml) were incubated in medium alone or in medium with mAbs (1 μg/ml) in a 96-well plate (Costar). After a 2-h incubation, cells were photographed with an inverted light microscope (Leica DM IL) equipped with a Leica DFC295 video camera. The mean aggregate size of three independent fields of view was quantified in ImageJ (66) by converting each image to binary and using the “analyze particles” function to measure the size of each aggregate in the field. Results are presented as mean ± S.D. of mean aggregate size over the three fields.

For inhibition experiments, cells were pre-incubated with 10 μm C_6_-ceramide or an equivalent volume of solvent (DMSO) only for 1 h at 37 °C before addition of the mAbs (42). All results shown are representative of three separate experiments.

##### Molecular Modeling

To model the position of 3FN9,10 bound to the headpiece of α5β1, the superposition of the crystal structures of the RGD-bound α5β1 (Protein Data Bank code 4WK4, with RGD peptide removed) (18) and the αVβ3–3FN10 complex (Protein Data Bank code 4MMX) (49) was generated using PyMOL (version 1.7.2.3; Schrödinger, LLC). The NMR structure of 3FN9,10 (Protein Data Bank code 2MFN; model 10) (50) was docked onto α5β1 by alignment with 3FN10 of the αVβ3–3FN10 complex. The obminimize function in Open Babel (version 2.3.1) (67) was used to confirm geometry optimization and energy minimization for the aligned molecules. Images were rendered in PyMOL and assembled using Illustrator (version CS6; Adobe). A video representation of the model was generated in PyMOL.

##### Cell Spreading Assays

HT-1080 fibrosarcoma cells were maintained in complete Dulbecco's modified Eagle's medium supplemented with 2 mm glutamine and 10% fetal bovine serum. Wells of a 96-well plate (Costar) were coated with 50K (2 μg/ml in Dulbecco's PBS) for 60 min at room temperature, and then sites on the plastic for nonspecific cell adhesion were blocked for 30 min at room temperature with 100 μl of 10 mg/ml heat-denatured BSA. HT-1080 cells were detached with 0.05% trypsin, 0.02% EDTA, resuspended to 5 × 10^5^/ml in serum-free Dulbecco's minimal essential medium with 25 mm HEPES, and allowed to recover for 10 min at 37 °C. Cells were then added to the wells and allowed to spread for 60 min in a humidified atmosphere of 5% CO_2_ at 37 °C. mAbs were then added to wells to a final concentration of 10 μg/ml, and the cells were incubated for a further 60 min. The cells were then fixed with 3% glutaraldehyde, and the degree of spreading was quantified using phase-contrast microscopy. Each data point (mean ± S.D.) was obtained by counting three sets of 100 cells/well from a number of randomly selected fields. In a parallel assay, mAbs were added to the cells at the same time as plating on 50K. No cell spreading was observed on wells coated only with heat-denatured BSA.

## Author Contributions

A. P. M. performed the SPR, ligand inhibition, and competitive ELISA experiments, designed the project, and wrote most of the manuscript. J. A. A. performed the SDS-PAGE, cell aggregation assays, the M287V mutant FACS analysis, and the cell spreading assays. A. B. performed the molecular modeling and helped with figure preparation. Y. T. performed the Lia1/2 epitope mapping experiments. T. A. J., M. J. H., and the other authors reviewed the results and contributed to manuscript preparation.

## Supplementary Material

Supplemental Data

## Footnotes

3 The abbreviations used are:

MIDAS: metal ion-dependent adhesion site
50K: 50-kDa fragment of fibronectin (3FN6–10)
IFC: integrin-fibronectin complex
mu IgG: mouse IgG
RB: running buffer
cRGD: cyclic GCRGDSPCG
SPR: surface plasmon resonance.

## References

[1] Hynes R. O. (2002) Integrins: bidirectional, allosteric signalling machines. Cell 110, 673–687 12297042 10.1016/s0092-8674(02)00971-6

[2] Goodman S. L., and Picard M. (2012) Integrins as therapeutic targets. Trends Pharmacol. Sci. 33, 405–412 22633092 10.1016/j.tips.2012.04.002

[3] Blandin A. F., Renner G., Lehmann M., Lelong-Rebel I., Martin S., and Dontenwill M. (2015) β1 integrins as therapeutic targets to disrupt hallmarks of cancer. Front. Pharmacol. 6, 279 26635609 10.3389/fphar.2015.00279 PMC4656837

[4] Giordano A., Musumeci G., D'Angelillo A., Rossini R., Zoccai G. B., Messina S., Coscioni E., Romano S., and Romano M. F. (2016) Effects of glycoprotein IIb/IIIa antagonists: anti platelet aggregation and beyond. Curr. Drug Metab. 17, 194–203 26652157 10.2174/1389200217666151211121112

[5] Rice G. P., Hartung H. P., and Calabresi P. A. (2005) Anti-α4 integrin therapy for multiple sclerosis: mechanisms and rationale. Neurology 64, 1336–1342 15851719 10.1212/01.WNL.0000158329.30470.D0

[6] Cherry L. N., Yunker N. S., Lambert E. R., Vaughan D., and Lowe D. K. (2015) Vedolizumab: an α4β7 integrin antagonist for ulcerative colitis and Crohn's disease. Ther. Adv. Chronic Dis. 6, 224–233 26336591 10.1177/2040622315586970 PMC4549690

[7] Hersey P., Sosman J., O'Day S., Richards J., Bedikian A., Gonzalez R., Sharfman W., Weber R., Logan T., Buzoianu M., Hammershaimb L., Kirkwood J. M., and Etaracizumab Melanoma Study Group. (2010) A randomized phase 2 study of etaracizumab, a monoclonal antibody against integrin αVβ3 + or − dacarbazine in patients with stage IV metastatic melanoma. Cancer 116, 1526–1534 20108344 10.1002/cncr.24821

[8] Mateo J., Berlin J., de Bono J. S., Cohen R. B., Keedy V., Mugundu G., Zhang L., Abbattista A., Davis C., Gallo Stampino C., and Borghaei H. (2014) A first-in-human study of the anti-α5β1 integrin monoclonal antibody PF-04605412 administered intravenously to patients with advanced solid tumors. Cancer Chemother. Pharmacol. 74, 1039–1046 25212537 10.1007/s00280-014-2576-8 PMC4209234

[9] Heidenreich A., Rawal S. K., Szkarlat K., Bogdanova N., Dirix L., Stenzl A., Welslau M., Wang G., Dawkins F., de Boer C. J., and Schrijvers D. (2013) A randomized, double-blind, multicenter, phase 2 study of a human monoclonal antibody to human αV integrins (intetumumab) in combination with docetaxel and prednisone for the first-line treatment of patients with metastatic castration-resistant prostate cancer. Ann. Oncol. 24, 329–336 23104724 10.1093/annonc/mds505

[10] Xiong J. P., Stehle T., Diefenbach B., Zhang R., Dunker R., Scott D. L., Joachimiak A., Goodman S. L., and Arnaout M. A. (2001) Crystal structure of the extracellular segment of integrin αVβ3. Science 294, 339–345 11546839 10.1126/science.1064535 PMC2885948

[11] Xiao T., Takagi J., Coller B. S., Wang J. H., and Springer T. A. (2004) Structural basis for allostery in integrins and binding to fibrinogen-mimetic therapeutics. Nature 432, 59–67 15378069 10.1038/nature02976 PMC4372090

[12] Xiong J.-P., Stehle T., Zhang R., Joachimiak A., Frech M., Goodman S. L., and Arnaout M. A. (2002) Crystal structure of the extracellular segment of integrin αVβ3 in complex with an Arg-Gly-Asp ligand. Science 296, 151–155 11884718 10.1126/science.1069040

[13] Irie A., Kamata T., and Takada Y. (1997) Multiple loop structures critical for ligand binding of the integrin α4 subunit in the upper face of the β-propeller model. Proc. Natl. Acad. Sci. U.S.A. 94, 7198–7203 9207068 10.1073/pnas.94.14.7198 PMC23791

[14] Kamata T., Tieu K. K., Irie A., Springer T. A., and Takada Y. (2001) Amino acid residues in the αIIb subunit that are critical for ligand binding to integrin αIIbβ3 are clustered in the β-propeller model. J. Biol. Chem. 276, 44275–44283 11557768 10.1074/jbc.M107021200

[15] Mould A. P., Askari J. A., Aota Si, Yamada K. M., Irie A., Takada Y., Mardon H. J., and Humphries M. J. (1997) Defining the topology of integrin α5β1-fibronectin interactions using inhibitory anti-α5 and anti-β1 monoclonal antibodies–evidence that the synergy sequence of fibronectin is recognized by the NH_2_-terminal repeats of the α5 subunit. J. Biol. Chem. 272, 17283–17292 9211865 10.1074/jbc.272.28.17283

[16] Mould A. P., Koper E. J., Byron A., Zahn G., and Humphries M. J. (2009) Mapping the ligand-binding pocket of integrin α5β1 using a gain-of-function approach. Biochem. J. 424, 179–189 19747169 10.1042/BJ20090992 PMC3329623

[17] Nagae M., Re S., Mihara E., Nogi T., Sugita Y., and Takagi J. (2012) Crystal structure of α5β1 integrin ectodomain: atomic details of the fibronectin receptor. J. Cell Biol. 197, 131–140 22451694 10.1083/jcb.201111077 PMC3317794

[18] Xia W., and Springer T. A. (2014) Metal ion and ligand binding of integrin α5β1. Proc. Natl. Acad. Sci. U.S.A. 111, 17863–17868 25475857 10.1073/pnas.1420645111 PMC4273411

[19] Aota S., Nomizu M., and Yamada K. M. (1994) The short amino acid sequence Pro-His-Ser-Arg-Asn in human fibronectin enhances cell-adhesive function. J. Biol. Chem. 269, 24756–24761 7929152

[20] Luo B. H., Carman C. V., and Springer T. A. (2007) Structural basis of integrin regulation and signaling. Annu. Rev. Immunol. 25, 619–647 17201681 10.1146/annurev.immunol.25.022106.141618 PMC1952532

[21] Askari J. A., Buckley P. A., Mould A. P., and Humphries M. J. (2009) Linking integrin conformation to function. J. Cell Sci. 122, 165–170 19118208 10.1242/jcs.018556 PMC2714414

[22] Zhu J., Zhu J., and Springer T. A. (2013) Complete integrin headpiece opening in eight steps. J. Cell Biol. 201, 1053–1068 23798730 10.1083/jcb.201212037 PMC3691460

[23] Mould A. P., Askari J. A., Barton S., Kline A. D., McEwan P. A., Craig S. E., and Humphries M. J. (2002) Integrin activation involves a conformational change in the α1 helix of the β subunit A-domain. J. Biol. Chem. 277, 19800–19805 11893752 10.1074/jbc.M201571200

[24] Mould A. P., Barton S. J., Askari J. A., McEwan P. A., Buckley P. A., Craig S. E., and Humphries M. J. (2003) Conformational changes in the integrin βA domain provide a mechanism for signal transduction via hybrid domain movement. J. Biol. Chem. 278, 17028–17035 12615914 10.1074/jbc.M213139200

[25] Barton S. J., Travis M. A., Askari J. A., Buckley P. A., Craig S. E., Humphries M. J., and Mould A. P. (2004) Novel activating and inactivating mutations in the integrin β1 subunit A domain. Biochem. J. 380, 401–407 14967067 10.1042/BJ20031973 PMC1224172

[26] Zhang C., Liu J., Jiang X., Haydar N., Zhang C., Shan H., and Zhu J. (2013) Modulation of integrin activation and signaling by α1/α1′-helix unbending at the junction. J. Cell Sci. 126, 5735–5747 24144695 10.1242/jcs.137828

[27] Burrows L., Clark K., Mould A. P., and Humphries M. J. (1999) Fine mapping of inhibitory anti-α5 monoclonal antibody epitopes that differentially affect integrin-ligand binding. Biochem. J. 344, 527–533 10567237 PMC1220672

[28] Humphries M. J., Symonds E. J., and Mould A. P. (2003) Mapping functional residues onto integrin crystal structures. Curr. Opin. Struct. Biol. 13, 236–243 12727518 10.1016/s0959-440x(03)00035-6

[29] Nishimichi N., Kawashima N., and Yokosaki Y. (2015) Epitopes in α8β1 and other RGD-binding integrins delineate classes of integrin-blocking antibodies and major binding loops in α subunits. Sci. Rep. 5, 13756 26349930 10.1038/srep13756 PMC4563375

[30] Mahalingam B., Van Agthoven J. F., Xiong J. P., Alonso J. L., Adair B. D., Rui X., Anand S., Mehrbod M., Mofrad M. R., Burger C., Goodman S. L., and Arnaout M. A. (2014) Atomic basis for the species-specific inhibition of αV integrins by mAb 17E6 is revealed by the crystal structure of αVβ3 ectodomain-17E6 Fab complex. J. Biol. Chem. 289, 13801–13809 24692540 10.1074/jbc.M113.546929 PMC4022854

[31] Yu Y., Schürpf T., and Springer T. A. (2013) How natalizumab binds and antagonizes α4 integrins. J. Biol. Chem. 288, 32314–32325 24047894 10.1074/jbc.M113.501668 PMC3820868

[32] Takada Y., and Puzon W. (1993) Identification of a regulatory region of integrin β1 subunit using activating and inhibiting antibodies. J. Biol. Chem. 268, 17597–17601 7688727

[33] Byron A., Humphries J. D., Askari J. A., Craig S. E., Mould A. P., and Humphries M. J. (2009) Anti-integrin monoclonal antibodies. J. Cell Sci. 122, 4009–4011 19910492 10.1242/jcs.056770 PMC3329622

[34] Yu Y., Zhu J., Mi L. Z., Walz T., Sun H., Chen J.-F., and Springer T. A. (2012) Structural specializations of α4β7, an integrin that mediates rolling adhesion. J. Cell Biol. 196, 131–146 22232704 10.1083/jcb.201110023 PMC3255974

[35] Mould A. P., Craig S. E., Byron S. K., Humphries M. J., and Jowitt T. A. (2014) Disruption of integrin-fibronectin complexes by allosteric but not ligand-mimetic inhibitors. Biochem. J. 464, 301–313 25333419 10.1042/BJ20141047

[36] Reardon D. A., and Cheresh D. (2011) Cilengitide: a prototypic integrin inhibitor for the treatment of glioblastoma and other malignancies. Genes Cancer 2, 1159–1165 22866207 10.1177/1947601912450586 PMC3411133

[37] Mason W. P. (2015) End of the road: confounding results of the CORE trial terminate the arduous journey of cilengitide for glioblastoma. Neuro. Oncol. 17, 634–635 25681307 10.1093/neuonc/nov018 PMC4482863

[38] Coe A. P., Askari J. A., Kline A. D., Robinson M. K., Kirby H., Stephens P. E., and Humphries M. J. (2001) Generation of a minimal α5β1 integrin-Fc fragment. J. Biol. Chem. 276, 35854–35866 11389148 10.1074/jbc.M103639200

[39] Clark K., Pankov R., Travis M. A., Askari J. A., Mould A. P., Craig S. E., Newham P., Yamada K. M., and Humphries M. J. (2005) A specific α5β1-integrin conformation promotes directional integrin translocation and fibronectin matrix formation. J. Cell Sci. 118, 291–300 15615773 10.1242/jcs.01623 PMC3329624

[40] Mould A. P., Travis M. A., Barton S. J., Hamilton J. A., Askari J. A., Craig S. E., Macdonald P. R., Kammerer R. A., Buckley P. A., and Humphries M. J. (2005) Evidence that monoclonal antibodies directed against the integrin β subunit plexin/semaphorin/integrin domain stimulate function by inducing receptor extension. J. Biol. Chem. 280, 4238–4246 15557320 10.1074/jbc.M412240200 PMC3328395

[41] Mould A. P., Akiyama S. K., and Humphries M. J. (1996) The inhibitory anti-β1 integrin monoclonal antibody 13 recognizes an epitope that is attenuated by ligand occupancy. Evidence for allosteric inhibition of integrin function. J. Biol. Chem. 271, 20365–20374 8702772 10.1074/jbc.271.34.20365

[42] Lee Y. G., Lee J., and Cho J. Y. (2010) Cell-permeable ceramides act as novel regulators of U937 cell-cell adhesion mediated by CD29, CD98, and CD147. Immunobiology 215, 294–303 19576658 10.1016/j.imbio.2009.05.009

[43] Campanero M. R., Pulido R., Ursa M. A., Rodríguez-Moya M., de Landázuri M. O., and Sánchez-Madrid F. (1990) An alternative leukocyte homotypic adhesion mechanism, LFA-1/ICAM-1 independent, triggered through the human VLA-4 integrin. J. Cell Biol. 110, 2157–2165 1693625 10.1083/jcb.110.6.2157 PMC2116145

[44] Campanero M. R., Arroyo A. G., Pulido R., Ursa A., de Matías M. S., Sánchez-Mateos P., Kassner P. D., Chan B. M., Hemler M. E., Corbí A. L., de Landazuri M. O., and Sanchez-Madrid E. (1992) Functional role of α2β1 and α4β1 integrins in leukocyte intercellular adhesion induced through the common β1 subunit. Eur. J. Immunol. 22, 3111–3119 1446704 10.1002/eji.1830221213

[45] Shen C. X., Stewart S., Wayner E., Carter W., and Wilkins J. (1991) Antibodies to different members of the β1 (CD29) integrins induce homotypic and heterotypic cellular aggregation. Cell. Immunol. 138, 216–228 1717163 10.1016/0008-8749(91)90146-3

[46] Muñoz M., Serrador J., Sánchez-Madrid F., and Teixidó J. (1996) A region of the integrin VLA α4 subunit involved in homotypic cell aggregation and in fibronectin but not vascular cell adhesion molecule-1 binding. J. Biol. Chem. 271, 2696–2702 8576243 10.1074/jbc.271.5.2696

[47] Wilkins J. A., Li A., Ni H., Stupack D. G., and Shen C. (1996) Control of β1 integrin function. Localization of stimulatory epitopes. J. Biol. Chem. 271, 3046–3051 8621699

[48] Cho J. Y., Fox D. A., Horejsi V., Sagawa K., Skubitz K. M., Katz D. R., and Chain B. (2001) The functional interactions between CD98, β1-integrins, and CD147 in the induction of U937 homotypic aggregation. Blood 98, 374–382 11435306 10.1182/blood.v98.2.374

[49] Van Agthoven J. F., Xiong J. P., Alonso J. L., Rui X., Adair B. D., Goodman S. L., and Arnaout M. A. (2014) Structural basis for pure antagonism of integrin αVβ3 by a high-affinity form of fibronectin. Nat. Struct. Mol. Biol. 21, 383–388 24658351 10.1038/nsmb.2797 PMC4012256

[50] Copié V., Tomita Y., Akiyama S. K., Aota S., Yamada K. M., Venable R. M., Pastor R. W., Krueger S., and Torchia D. A. (1998) Solution structure and dynamics of linked cell attachment modules of mouse fibronectin containing the RGD and synergy regions: comparison with the human fibronectin crystal structure. J. Mol. Biol. 277, 663–682 9533887 10.1006/jmbi.1998.1616

[51] Beglova N., Blacklow S. C., Takagi J., and Springer T. A. (2002) Cysteine-rich module structure reveals a fulcrum for integrin rearrangement upon activation. Nat. Struct. Biol. 9, 282–287 11896403 10.1038/nsb779

[52] Zhang P., Fu C., Hu Y., Dong C., Song Y., and Song E. (2015) C6-ceramide nanoliposome suppresses tumor metastasis by eliciting PI3K and PKCζ tumor-suppressive activities and regulating integrin affinity modulation. Sci. Rep. 5, 9275 25792190 10.1038/srep09275 PMC4366857

[53] Pike K. A., Kulkarni S., and Pawson T. (2011) Immature T-cell clustering and efficient differentiation require the polarity protein Scribble. Proc. Natl. Acad. Sci. U.S.A. 108, 1116–1121 21189299 10.1073/pnas.1018224108 PMC3024664

[54] Mould A. P., Symonds E. J., Buckley P. A., Grossmann J. G., McEwan P. A., Barton S. J., Askari J. A., Craig S. E., Bella J., and Humphries M. J. (2003) Structure of an integrin-ligand complex deduced from solution x-ray scattering and site-directed mutagenesis. J. Biol. Chem. 278, 39993–39999 12871973 10.1074/jbc.M304627200

[55] Takagi J., Strokovich K., Springer T. A., and Walz T. (2003) Structure of integrin α5β1 in complex with fibronectin. EMBO J. 22, 4607–4615 12970173 10.1093/emboj/cdg445 PMC212714

[56] Crump J. G., Swartz M. E., and Kimmel C. B. (2004) An integrin-dependent role of pouch endoderm in hyoid cartilage development. PLoS Biol. 2, E244 15269787 10.1371/journal.pbio.0020244 PMC479042

[57] Orlando R. A., and Cheresh D. A. (1991) Arginine-glycine-aspartic acid binding leading to molecular stabilization between integrin αVβ3 and its ligand. J. Biol. Chem. 266, 19543–19550 1717468

[58] Müller B., Zerwes H. G., Tangemann K., Peter J., and Engel J. (1993) Two-step binding mechanism of fibrinogen to αIIbβ3 integrin reconstituted into planar lipid bilayers. J. Biol. Chem. 268, 6800–6808 8454652

[59] Peerschke E. I. (1999) Maintenance of GPIIb–IIIa avidity supporting “irreversible” fibrinogen binding is energy-dependent. J. Lab. Clin. Med. 134, 398–404 10521087 10.1016/s0022-2143(99)90155-5

[60] Kasirer-Friede A., Kang J., Kahner B., Ye F., Ginsberg M. H., and Shattil S. J. (2014) ADAP interactions with talin and kindlin promote platelet integrin αIIbβ3 activation and stable fibrinogen binding. Blood 123, 3156–3165 24523237 10.1182/blood-2013-08-520627 PMC4023421

[61] Dickreuter E., Eke I., Krause M., Borgmann K., van Vugt M. A., and Cordes N. (2016) Targeting of β1 integrins impairs DNA repair for radiosensitization of head and neck cancer cells. Oncogene 35, 1353–1362 26073085 10.1038/onc.2015.212

[62] Kimura H., Tome Y., Momiyama M., Hayashi K., Tsuchiya H., Bouvet M., and Hoffman R. M. (2012) Imaging the inhibition by anti-β1 integrin antibody of lung seeding of single osteosarcoma cells in live mice. Int. J. Cancer 131, 2027–2033 22323248 10.1002/ijc.27475

[63] Park C. C., Zhang H. J., Yao E. S., Park C. J., and Bissell M. J. (2008) β1 integrin inhibition dramatically enhances radiotherapy efficacy in human breast cancer xenografts. Cancer Res. 68, 4398–4405 18519702 10.1158/0008-5472.CAN-07-6390 PMC3719863

[64] Mould A. P., Garratt A. N., Puzon-McLaughlin W., Takada Y., and Humphries M. J. (1998) Regulation of integrin function: evidence that bivalent-cation-induced conformational changes lead to the unmasking of ligand-binding sites within integrin α5β1. Biochem. J. 331, 821–828 9560310 10.1042/bj3310821 PMC1219423

[65] Parsons M., Messent A. J., Humphries J. D., Deakin N. O., and Humphries M. J. (2008) Quantification of integrin receptor agonism by fluorescence lifetime imaging. J. Cell Sci. 121, 265–271 18216331 10.1242/jcs.018440 PMC3328206

[66] Collins T. J. (2007) ImageJ for microscopy. BioTechniques 43, S25–S30 10.2144/000112517 17936939

[67] O'Boyle N. M., Banck M., James C. A., Morley C., Vandermeersch T., and Hutchison G. R. (2011) Open Babel: An open chemical toolbox. J. Cheminform. 3, 33 21982300 10.1186/1758-2946-3-33 PMC3198950

[68] Mould A. P., Burrows L., and Humphries M. J. (1998) Identification of amino acid residues that form part of the ligand-binding pocket of integrin α5β1. J. Biol. Chem. 273, 25664–25672 9748233 10.1074/jbc.273.40.25664

[69] Humphries J. D., Askari J. A., Zhang X. P., Takada Y., Humphries M. J., and Mould A. P. (2000) Molecular basis of ligand recognition by integrin α5β1. II. Specificity of Arg-Gly-Asp binding is determined by Trp157 of the α subunit. J. Biol. Chem. 275, 20337–20345 10764747 10.1074/jbc.M000568200

[70] Askari J. A., Tynan C. J., Webb S. E., Martin-Fernandez M. L., Ballestrem C., and Humphries M. J. (2010) Focal adhesions are sites of integrin extension. J. Cell Biol. 188, 891–903 20231384 10.1083/jcb.200907174 PMC2845069

[71] Askari J. A. (2006) Conformational Changes in Integrin α5β1. Ph.D. thesis, University of Manchester, UK

[72] Ni H., and Wilkins J. A. (1998) Localisation of a novel adhesion blocking epitope on the human β1 integrin chain. Cell Adhes. Commun. 5, 257–271 9762467 10.3109/15419069809040296

