# Supplementary material for: Ligand-induced Epitope Masking: DISSOCIATION OF INTEGRIN α5β1-FIBRONECTIN COMPLEXES ONLY BY MONOCLONAL ANTIBODIES WITH AN ALLOSTERIC MODE OF ACTION
Source: J Biol Chem. 2016 Aug 2;291(40):20993–1007. doi: 10.1074/jbc.M116.736942 (PMC5076510; doi:10.1074/jbc.M116.736942)
Supplement: Supplemental Data [file supp_291_40_20993__index.html]

Ligand-induced epitope masking. Dissociation of integrin α5β1-fibronectin complexes only by monoclonal antibodies with an allosteric mode of action. — Ligand-induced Epitope Masking — Ligand Occupancy Causes Masking of Epitopes in α5β1 — Supplemental Data 

# Ligand-induced Epitope Masking

## Supplemental Data

- Supplemental video S1 (.mov, 4.1 MB) - movie
